# stpm: an R package for stochastic process model

**DOI:** 10.1186/s12859-017-1538-7

**Published:** 2017-02-23

**Authors:** Ilya Y. Zhbannikov, Konstantin Arbeev, Igor Akushevich, Eric Stallard, Anatoliy I. Yashin

**Affiliations:** 1 Biodemography of Aging Research Unit (BARU) at Social Science Research Institute, Duke University, 2024 W. Main St., Durham, Box 90420, 27705 NC USA; 2 Duke Population Research Institute, Duke University, Durham, Box 90989, 27708-0989 NC USA

**Keywords:** Stochastic process model, Quadratic hazard, Longitudinal data, Life tables, Risk factors

## Abstract

**Background:**

The Stochastic Process Model (SPM) represents a general framework for modeling the joint evolution of repeatedly measured variables and time-to-event outcomes observed in longitudinal studies, i.e., SPM relates the stochastic dynamics of variables (e.g., physiological or biological measures) with the probabilities of end points (e.g., death or system failure). SPM is applicable for analyses of longitudinal data in many research areas; however, there are no publicly available software tools that implement this methodology.

**Results:**

We developed an R package stpm for the SPM-methodology. The package estimates several versions of SPM currently available in the literature including discrete- and continuous-time multidimensional models and a one-dimensional model with time-dependent parameters. Also, the package provides tools for simulation and projection of individual trajectories and hazard functions.

**Conclusion:**

In this paper, we present the first software implementation of the SPM-methodology by providing an R package stpm, which was verified through extensive simulation and validation studies. Future work includes further improvements of the model. Clinical and academic researchers will benefit from using the presented model and software. The R package stpm is available as open source software from the following links: https://cran.r-project.org/package=stpm(stable version) or https://github.com/izhbannikov/spm(developer version).

**Electronic supplementary material:**

The online version of this article (doi:10.1186/s12859-017-1538-7) contains supplementary material, which is available to authorized users.

## Background

A plethora of approaches to the joint analysis of longitudinal and time-to-event (survival) data have been developed the in last few decades, see ([1], Ch. 11) and [2, 3]. For example, joint longitudinal-survival models analyze the joint behavior of the process describing physiological variables (i.e. “longitudinal” process) and time-to-event (“survival”) process, see [4–7] and R package JM[8], lcmm[9]. These models usually represent the hazard in the form of the Cox proportional hazards model [10] and dynamics of longitudinal variable as a mixed-effects model [11]. Also, random processes (e.g., Ornstein-Uhlenbeck or Gaussian processes) can be used in order to handle random fluctuations of individual measurements around the population average [12, 13].

There are also extensions, such as accelerated failure time or additive hazard models [14, 15] (see, e.g. R - package JM), which can be applied if the Cox proportional hazards assumption is violated.

However, an important challenge to consider in the context of bioinformatic studies is integration of biological knowledge and theories with statistical and computational methods and algorithms. One of possible approaches to integrate biological concepts and statistical models is based on the quadratic hazard models (also known alternatively as Stochastic Process Models, SPM) which were first introduced several decades ago [16–18]. Such models were recently modified [19, 20] to incorporate several conceptual mechanisms with clear biological interpretation (such as homeostatic regulation, allostatic adaptation, stress resistance, adaptive capacity and physiological norms) relevant in the context of research on aging. Incorporation of available knowledge about regularities of aging-related changes developing in the human body into the model structure allows for addressing fundamental problems of aging dealing with age-related declines in stress resistance and adaptive capacity, changes in resilience and physiological norm, accumulation of allostatic load, etc. [20]. Importantly, these models permit evaluating all these mechanisms indirectly from longitudinal trajectories of biomarkers and data on mortality or onset of diseases when measurements of relevant variables representing the respective biological processes are not available in the data. Thus SPM provides an example of a successful attempt to analytically link biological knowledge about aging-related processes developing in the human body with changes in mortality risk using a compact and convenient mathematical framework.

The idea of SPM was first described in [16]. The theoretical background of the models with survival functions affected by stochastic covariates was also presented in [17, 21–23]. Later the methodology was extended in several publications, e.g. [19, 24–29]. The SPM links the dynamics of stochastic variables represented by multivariable autoregressive or stochastic differential equations with hazard rates described as quadratic functions of the state variables. The choice of the quadratic hazard function (also known as U- or J- shaped hazard function) is justified empirically based on many epidemiological observations for various biomarkers (see, e.g., [30–35]). The minimum of a quadratic function (or paraboloid in the multivariable case) is a point (or domain) in the variable state space, which corresponds to the optimal system status (e.g., the “normal” health status) with the minimal value of mortality risk at a specific time (or age). An important component of the SPM is the observational plan which characterizes how dynamic variables affecting mortality risk were measured in a longitudinal study.

We note also that such models have a much broader range of applications in many areas beyond research on aging capitalizing on their strength of using the stochastic dynamics of variables which may better describe the reality in many applications. Many publications demonstrated the high relevance of using the SPM in joint analyses of longitudinal measurements of various variables and time-to-event outcomes. However, until now, there were no publicly available software tools that implement this kind of analysis.

In this paper we present the R package, stpm, the first publicly available set of utilities which implements the SPM methodology in three specific cases covering analyses most frequently used in practice and, therefore, constituting a general framework for studying and modeling survival (censored) traits depending on random trajectories (stochastic paths) of variables.

## Implementation

### Model forms

There are two forms of the SPM that have been developed recently stemming from the original works by Woodbury, Manton, Yashin, Stallard and colleagues in 1970–1980’s: (i) discrete-time stochastic process model, assuming fixed time intervals between subsequent observations, initially developed by Woodbury, Manton et al. [16, 23] and further developed by Akushevich et al. [24]; (ii) and continuous-time model, proposed in Yashin et al. [17, 22] (and later extended in [19]), which can handle arbitrary time intervals. The 2007 version [19] specifies the components of the model tailored to applications in aging research which can still be used in a more general context.

In the R package stpm we implemented the models of type i and ii with time-independent coefficients, which can handle one or more variables (dimensions). In addition, we implemented a one-dimensional case (when one physiological variable/covariate is used) with time-dependent coefficients of the model in [19]. Below we briefly describe the types of stochastic process models implemented in stpm.

### Discrete-time SPM

The model [23, 24] assumes fixed time intervals between consecutive observations. In this model, **Y**(*t*) (a *k*×1 matrix of the values of covariates, where *k* is the number of covariates considered) and *μ*(*t*,**Y**(*t*)) (the hazard rate) have the following form:

1 $$  \begin{aligned} \text{} & \mathbf{Y}(t+1) = \mathbf{u} + \mathbf{R} \mathbf{Y}(t) + \mathbf{\epsilon} \\ & \mu (t, \mathbf{Y}(t)) = [\mu_{0} + \mathbf{b} \mathbf{Y}(t) + \mathbf{Y}(t)^{*} \mathbf{Q} \mathbf{Y}(t)] e^{\theta t} \end{aligned}  $$

Coefficients **u** (a *k*×1 matrix, where *k* is a number of covariates), **R** (a *k*×*k* matrix), *μ*_0_, **b** (a 1×*k* matrix), **Q** (a *k*×*k* matrix) are assumed to be constant in the particular implementation of this model in the R package stpm. **ε** contains normally-distributed random residuals, a *k*×1 matrix. The symbol “*” denotes transpose operation. *θ* is a parameter to be estimated along with other parameters (**u**, **R**, *μ*_0_, **b**, **Q**).

These coefficients are then estimated directly from (i) linear auto-regression (**Y**(*t*+1)=**u**+**R** **Y**(*t*)+**ε**), where **Y**(*t*) is empirically-observed for those subjects that are alive at time *t* and **Y**(*t*+1) is the value of **Y**(*t*) at time *t*+1; (ii) using a generalized linear model with family *Binomial* and link *Log*.

### Continuous-time SPM

In the specification of the SPM described in the 2007 paper by Yashin and collegaues [19] the stochastic differential equation describing the age dynamics of a covariate is:

2 $$  d\mathbf{Y}(t)= \mathbf{a}(t)(\mathbf{Y}(t) -\mathbf{f}_{1}(t))dt + \mathbf{b}(t)d\mathbf{W}(t), \mathbf{Y}(t=t_{0})  $$

In this equation, **Y**(*t*) (a *k*×1 matrix) is the value of a particular covariate at time (age) *t*. **f**_1_(*t*) (a *k*×1 matrix) corresponds to the long-term mean value of the stochastic process **Y**(*t*) which describes a trajectory of individual covariates influenced by different factors represented by a random Wiener process **W**(*t*). Coefficient **a**(*t*) (a *k*×*k* matrix) is a negative feedback coefficient which characterizes the rate at which the process reverts to its mean. In the area of research on aging, **f**_1_(*t*) represents the mean allostatic trajectory and **a**(*t*) represents the adaptive capacity of the organism. Coefficient **b**(*t*) (a *k*×1 matrix) characterizes the strength of the random disturbances from the Wiener process **W**(*t*).

The following function *μ*(*t*,**Y**(*t*)) represents the hazard rate:

3 $$  \mu(t, \mathbf{Y}(t)) = \mu_{0}(t) + (\mathbf{Y}(t) - \mathbf{f}(t))^{*} \mathbf{Q}(t) (\mathbf{Y}(t) - \mathbf{f}(t))  $$

here *μ*_0_(*t*) is the baseline hazard, which corresponds to the risk when **Y**(*t*) follows its optimal trajectory; **f**(*t*) (a *k*×1 matrix) represents the optimal trajectory that minimizes the risk and **Q**(*t*) (a *k*×*k* matrix) models the sensitivity of the risk function to deviations from the norm.

In general, model coefficients **a**(*t*), **f**_1_(*t*), **Q**(*t*), **f**(*t*), **b**(*t*) and *μ*_0_(*t*) are time(age)-dependent. For example, the coefficient **a** can be assumed as (i) -0.05 (a constant, time-independent) or (ii) **a**(*t*)=**a**_0_+**b**_0_ *t* (time-dependent), in which **a**_0_ and **b**_0_ are unknown parameters to be estimated. The model can handle, in theory, any number of covariates.

In the implementation of the continuous-time SPM provided by the R package stpm, coefficients **a**, **f**_1_, **f**, **b**, *μ*_0_, **Q** are assumed to be time-independent. However, *μ*_0_ and **Q** from (3) can be multiplied by *e* ^*θt*^ (by user’s choice) and therefore are time-dependent: *μ*_0_(*t*)=*μ*_0_ *e* ^*θt*^, **Q**(*t*)=**Qe** ^*θt*^. If not, they are assumed to be time-independent along with the other coefficients. Then the maximum likelihood method is used to estimate parameters **a**, **f**_1_, **Q**, **f**, **b**, *μ*_0_,*θ* as described further.

#### Parameter estimation procedure

The parameter estimation procedure can be found, e.g., in [19] and here we briefly summarize it. As shown in [19], the likelihood function is:

4 $$ {}\begin{aligned} L = &\prod_{i=1}^{N} \prod_{j=1}^{n_{i}(\tau_{j})}{ (2\pi)^{-k/2} |\gamma^{i}\left(t^{i}_{j^{-}}\right)|^{-1/2} e^{-\frac{1}{2}\left(y^{i}_{t^{i}_{j}} - m^{i}\left(t^{i}_{j^{-}}\right)\right)^{*} \gamma^{i}\left(t^{i}_{j^{-}}\right)^{-1} \left(y^{i}_{t^{i}_{j}} - m^{i}\left(t^{i}_{j^{-}}\right)\right)} } \\ &\times \bar{\mu}^{i}(\tau_{i})^{\delta_{i}} e^{-\int_{0}^{\tau_{i}}{\bar{\mu}^{i}(u)du} } \end{aligned}  $$

5 $$ \begin{aligned} {} \bar{\mu}(u) =& \mu_{0}(u) + (\mathbf{m}(u) - \mathbf{f}(u))^{*} \times \mathbf{Q}(u) \times (\mathbf{m}(u) - \mathbf{f}(u))\\ &+ Tr(\mathbf{Q}(u) \times\mathbf{\gamma}(u)) \end{aligned}  $$

6 $$ {}\frac{dm(t)}{dt} = \mathbf{a}(t) (\mathbf{m}(t) - \mathbf{f_{1}}(t)) - 2 \mathbf{\gamma}(t) \mathbf{Q}(t) (\mathbf{m}(t) - \mathbf{f}(t)), m(0)  $$

7 $$ \begin{aligned} \frac{d\mathbf{\gamma}(t)}{dt} =& \mathbf{a}(t) \mathbf{\gamma}(t) + \mathbf{\gamma}(t) \mathbf{a}(t)^{*} + \mathbf{b}(t) \mathbf{b}(t)^{*} \\ &- 2 \mathbf{\gamma}(t) \mathbf{Q}(t) \mathbf{\gamma}(t), \gamma(0) \end{aligned}  $$

Here *L* is the likelihood; *i* denotes individual, *j* denotes observation for respective variable. In Eq. (5), (6), (7) we suppressed *i* and *j* for brevity. $\bar {\mu }(u)$ is the marginal hazard, presented in the survival function associated with the lifespan distribution $P(T > t) = exp(-\int _{0}^{t}{\bar {\mu }^{i}(u)du})$; *m*(0) and *γ*(0) are the mean and the variance/covariance matrix of the normal distribution of initial vector **Y**_0_=**Y**(*t*=*t*_0_) and the mean and the variance/covariance matrix of this distribution at age *t* are given by *m*(*t*) and *γ*(*t*), respectively; *Tr* denotes the trace of a matrix; $y^{i}_{t^{i}_{0}}$, $y^{i}_{t^{i}_{1}}$,..., $y^{i}_{t^{i}_{n_{i}}}$ denote the measurements of the process *Y*(*t*); *τ*_*i*_ is the lifespan (or age at censoring); *δ*_*i*_ is a censoring indicator, *m* ^*i*^(*t*) and *γ* ^*i*^(*t*) satisfy (6),(7) at the intervals $[t^{i}_{0}, t^{i}_{1})$, $[t^{i}_{1}, t^{i}_{2})$,..., $[t^{i}_{n_{i} - 1}, t^{i}_{n_{i}})$, $[t^{i}_{n_{i}}, \tau _{i})$ with the initial conditions $y^{i}_{t^{i}_{0}}$, $y^{i}_{t^{i}_{1}}$,..., $y^{i}_{t^{i}_{n_{i}}}$; $m^{i}\left (t^{i}_{j^{-}}\right) = {\lim }_{t \uparrow t^{i}_{j}}m^{i}(t)$, and $\gamma ^{i}(t^{i}_{j^{-}}) = {\lim }_{t \uparrow t^{i}_{j}}\gamma ^{i}(t)$.

We use available optimization methods from package nloptr to estimate the parameters of this model. By default we use the Nelder–Mead method [36].

The coefficient conversion between continuous- and discrete-time models is as follows (“c” and “d” denote continuous- and discrete-time models respectively; note: these equations can be used if the intervals between consecutive observations of discrete- and continuous-time models are equal; it is also required that matrices **a**_*c*_ and **Q**_*c,d*_ be full-rank matrices):

8 $$ \begin{array}{cc}& {\mathbf{Q}}_c={\mathbf{Q}}_d\\ {}{\mathbf{a}}_c={\mathbf{R}}_d- I(k)\\ {}{\mathbf{b}}_c=\boldsymbol{\Sigma} \\ {}{{\mathbf{f}}_1}_c=-{\mathbf{a}}_c^{-1}\times {\mathbf{u}}_d\\ {}{\mathbf{f}}_c=-0.5{\mathbf{b}}_d\times {\mathbf{Q}}_d^{-1}\\ {}{\mu_0}_c={\mu_0}_d-{\mathbf{f}}_c\times {\mathbf{Q}}_{\mathbf{c}}\times {\mathbf{f}}_c^{\ast}\\ {}{\theta}_c={\theta}_d\end{array} $$

where *k* is the number of covariates, which is equal to the model’s dimension and “*” denotes transpose operation; **Σ** is a *k*×1 matrix which contains the *s.d.*s of the corresponding residuals (residuals of a linear regression **Y**(*t*+1)=**u**+**R** **Y**(*t*)+**ε**; *s.d.* is a standard deviation), *I*(*k*) is an identity *k*×*k* matrix.

### Model with time-dependent coefficients

The two types of models described above assumes time-independent coefficients, i.e. coefficients are constant and one-dimensional through the lifetime. We also implemented a model in which the coefficients are time-dependent functions.

### Description of the R package stpm

The general workflow of parameter estimation in the stpm R package consists of (i) data preparation and (ii) model parameter estimation. A user can potentially avoid the data preparation stage but should maintain an appropriate data format as described below and in the package user manual. The package is available as open source software from the following link: https://cran.r-project.org/package=stpm (stable version) or https://github.com/izhbannikov/spm(developer version).

#### Input data

The input data consists of longitudinal follow-up data that needs to be presented in the form of a dataset in comma-separated or SAS formats. The dataset is a longitudinal data file in a *long* format (i.e. each record represents a single observation for a subject, therefore there are multiple rows per individual). An example is presented in Table 1.

**Table 1:** Example of longitudinal dataset

ID^a^	IndicatorDeath^b^	Age	AgeNext	DBP^c^	BMI
1	0	30	32	80	25.00
1	0	32	34	80	26.61
1	1	34	35.34	NA	NA
2	0	30	38	77	32.40
2	0	38	40	94	31.92
2	0	40	40.56	88	32.89
...	...	...	...	...	...
2	0	80	80.55	83	26.71
...					

^a^A subject identification number

^b^ IndicatorDeath shows that death occurred (IndicatorDeath=1) or did not occur (IndicatorDeath=0) between Age and AgeNext. Age for the next observation of the same individual must coincide with AgeNext of the current observation. AgeNext is a censoring age for the last observation.

^c^ DBP and BMI are measured at age Age and are diastolic blood pressure and body mass index. They are covariates. If some values of covariates are missing (but the subject is alive), they are imputed during the data preparation stage (see section “Data preparation”)

#### Data preparation

At the data preparation stage, the longitudinal dataset is preprocessed with the following command:



Here longdat.csv is a path to the longitudinal dataset (i.e., can be csv or SAS data file). Names of specific covariates can be explicitly mentioned:



In this case we mentioned two covariates: DBP, which is diastolic blood pressure, and BMI - body mass index. Therefore, only these covariates will be “prepared” for downstream analysis. By default the first three columns of data give individual id, censoring status, times of measurements, and the values of measured covariates are provided in the rest (see Tables 1, 2 and 3).

**Table 2:** Preprocessed table for discrete-time optimization. This table is used in the function spm_discrete(...)

id	case	t1	t2	DBP	DBP.next
1	0	78	80.00	74.70	75.37
1	0	80	82.00	75.37	72.14
1	0	82	84.00	72.14	67.03
1	1	84	85.34	67.03	71.22
2	0	30	32.00	80.00	80.49
2	0	32	34.00	80.49	88.20
2	0	34	36.00	88.20	89.36
....					
2	0	82	83.55	74.01	78.18
3	0	30	32.00	80.00	83.67
3	0	32	34.00	83.67	93.03
...

**Table 3:** Preprocessed table for continuous-time optimization. This table goes into function spm_continuous(...)

id	case		t1	t2	DBP DBP.next
1	0	76	77.03	73.68	71.70
1	0	77.03	78.11	71.70	73.20
....					
1	0	83.14	84.00	72.14	69.58
1	1	84.00	85.34	69.58	67.03
2	0	30.72	32.00	80.03	80.40
2	0	32	33.23	80.40	80.24
....					
2	0	79.80	81.57	69.84	74.01
2	0	81.57	83.55	74.01	78.18
3	0	31.42	32.91	79.48	80.50
3	0	32.91	33.79	80.50	81.83
....					

The output of prepare_data(...) function includes a list of two datasets for modeling data with arbitrary or fixed intervals. A dataset with fixed intervals is used in the package function spm_discrete(...) which implements discrete-time model; a dataset with arbitrary time intervals is used in the package function spm_continuous(...) for continuous-time model (theoretically, there might be missing values in this data set and the algorithm can impute them). Linear interpolation is used for the former case to provide values of covariates between predetermined (empirically-observed) time points. Tables 2 and 3 are examples of such data sets. Those tables contain no missing values.

#### Model parameter estimation

At the parameter estimation stage, the stpm R-package offers three SPM specifications: (a) discrete-time, multi-dimensional SPM [23, 24]; (b) continuous-time, multi-dimensional SPM [19]; (c) continuous-time, one-dimensional SPM with time-dependent user-defined coefficients [25]. The package’s central function spm(...) is used to estimate parameters from the model with different specifications and can be executed with the following command:



In this command: d.prep is a dataset (preprocessed data from function prepare_data(...)); model is a model type, the choices are: “discrete”, “continuous”, and “time-dependent.” For discrete and continuous model types, the output is a list with two subsets (parameters of these subsets are unambiguously related): (i) a set of estimated parameters [u, R, b, Sigma, Q, mu0, theta], see Eqs. (1); (ii) a set of estimated parameters [a, f1, Q, f, b, mu0, theta], see Eqs. (2, 3).

Output for SPM with time-dependent parameters gives estimates for parameters provided in formulas, which is a list of formulas that define the time-dependent parameters. If some parameter’s formulas were not explicitly indicated by a user in formulas then their defaults will be used and estimates will be given. The corresponding R-function to call this type of model is:



In this case the parameter formulas re-defines a. The model parameters not mentioned in the list formulas are constants (default). Initial values of parameters in formulas remaining for t=0 are estimated from the discrete-time model and initial values of parameters that define time dependence (e.g., a1 in the above example) are set to zero.

In the toy example below we summarize the data preparation and parameter estimation stages in a typical workflow. Datasets stored in longdat.csv are simulated data of two covariates (diastolic blood pressure, *DBP*, and body-mass index, *BMI*) estimated for 100 subjects. After this example we provide descriptions of the results.



p.discr.model, p.cont.model contain parameters estimated for discrete-time and continuous-time models. p.td.model contains parameters estimated for the SPM with time-dependent coefficients.

### Projection and simulation studies

The R package stpm also allows projection and data simulation with previously estimated or user-defined parameters. Projections are constructed for a cohort with normally distributed initial covariates. The results of the projections are (i) a dataset with individual projected values and (ii) a dataset with survival probabilities and age-specific means of state variables (covariates). An example of projection is:



The model.par here is a list of estimated model parameters from spm(...) function, 5000 is the number of individuals to simulate, 80 is the mean value of a covariate (in this case we have one-dimensional simulation). We present an example of simulation of 5,000 individuals: a data table and survival probabilities.



**Fig. 1:**
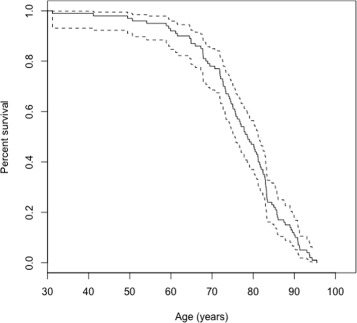
The Kaplan-Meier estimate (along with confidence intervals) of the survival function of one simulated dataset generated by the procedure described in “Simulation strategies” section

Here we first set the model parameters:



Then we call a simulation function spm_projection(...) in order to simulate data (we specify a starting age of 30 (tstart=30)). We also can see mean values of covariates for each age group (with a command data$stat$mean.by.age) and plot survival curves (see Fig. 1).

#### Simulation strategies

Simulation is needed for verification of the estimation procedure. Below we describe simulation strategies implemented in the R package stpm. All three models described above were verified through simulation studies.

To begin, a cohort of individuals at an initial time *t*_0_ is constructed. We construct individual trajectories as the solution of Eqs. (1), (2) for discrete- and continuous-time models using initial values of covariates, and random stopping (death) times. The initial values of covariates for all individuals in the cohort are simulated through sampling from the Gaussian distribution: $\mathbf {Y}(t=t_0) \sim N(f_{1}(t=t_{0}), {\sigma }_{0}^2)$, where *f*_1_(*t*=*t*_0_) is a value of function *f*_1_ at starting time *t*_0_ (both user-defined) and *σ*_0_ a standard deviation, user-defined (by default *σ*_0_=1 for any covariate).

Once we have the initial distribution of values of covariates for individuals, we then model trajectories in the multidimensional state space as follows:

- First, the conditional probability of survival for each individual is computed using the mortality rate *μ*(*t*,**Y**(*t*)) for the interval (*t*, *t* + *Δ* *t*): $S(t|\mathbf {Y}(t)) = e^{-\int _{t}^{t+\Delta t}\mu (s, \mathbf {Y}(s)) ds}$ (for continuous-time model) and *S*(*t*|**Y**(*t*))=*e* ^−*μ*(*t*,**Y**(*t*))*Δt*^ for discrete-time model.
- Each individual in the simulated cohort is deemed to survive or not, according to the probability *S*(*t*|**Y**(*t*)). To do that, a uniformly distributed random number *r* from the interval [0, 1] is generated. If *r*>*S*(*t*|**Y**(*t*)), the individual is assumed to have died, and the simulation of the corresponding individual trajectory stops at time *t*+*Δ* *t* (the time of death).
- Next, the covariate **Y**(*t*+*Δ* *t*) for a surviving individual is modeled using Eqs. (1) for discrete-time model or (2) for continuous-time model. The next observation time is modeled by adding *Δ* *t*, which is fixed for discrete-time model and arbitrary (*Δ* *t*=*step*+*unif*(−0.1*step*,0.1*step*) where parameter *step* is fixed and user-defined; by default *step*=1) for continuous-time model, to the current time *t*.
- If the age of a particular individual exceeded a maximum age tmax (user-defined, 105 by default), the individual is censored and trajectory simulation is stopped at time *t*+*Δ* *t* (a time of censoring). We also provided the possibility of censoring after achievement of *n* observations for a particular individual.

The whole process is repeated until all individuals have died or are censored.

### Validation

We conducted simulations of 100 follow-up datasets with discrete intervals (1 year) between the observations, with 5,000 of subjects in each dataset separately for one and two covariates. Separately, we simulated another set of 100 follow-up datasets with arbitrary intervals between observation (for continuous-time model, for one and two covariates). Trajectory projections were performed according to the methodologies described above. Finally, we performed simulation of 100 follow-up datasets for the model with time-dependent parameters and one covariate. For this model we set the parameter *f*_1_=*f*_1*a*_+*f*_1*b*_ *t*; other parameters were left as constants. Then we estimated all the parameters for discrete-, continuous-time and the model with time-dependent coefficients, for one and two covariates. The results are described in Discussion.

## Case study: application to the Framingham Heart Study Data

### Biological reasoning for the components of SPM [19]

In this case study we illustrate the application of SPM [19] in the context of biological questions in studies of aging. As we noted, one of the challenges in the context of bioinformatic studies is to incorporate biological concepts into statistical models. Understandably, representing biological mechanisms relevant to functioning of such complicated systems as the human organism in the framework of a mathematical or statistical model is a tremendous task. Nevetheless, one can try to represent in the model some basic components of the system under study. The SPM (its 2007 version, [19]) represents such an attempt to incorporate basic concepts in the field of research on aging in the framework of mathematical equations.

The first equation of the SPM (see eq. 2) represents the stochastic dynamics of biomarkers. The stochastic component of the model is an important part of the aging process [37], therefore, it is natural to use stochastic processes in the models of aging. One type of process which is relevant for describing biological processes in a living organism is the so-called *mean-reverting* stochastic process [38]. Such a process has a tendency to move to its equilibrium state (also called a long-term mean) and it can represent homeostatic regulation in the structure of the model (which is a critical property of the living organism). In reality, organisms function in a non-optimal environment, therefore the regulatory systems push it to a different sub-optimal state, which is known as the *allostatic state* [39]. Representation of the *mean allostatic state* in the SPM is another important illustration of inclusion of biological reasoning in statistical models for research on aging. The statistical concept of a negative feedback coefficient *a*(*t*) provides one more way to include biological concepts in the model. The coefficient *a*(*t*) controls how quickly the physiological trajectory reverts to its average and modulates the adaptive response rate of an organism to the stress factors. Such factors impact the biomarker trajectories so they deviate from their *normal* (optimal) states. For example, one research question could be to look at the age dynamics of adaptive capacity. The phenomenon of worsening adaptive capacity with age implies that more time is required for the values of biomarkers to return to the average allostatic state for older people in comparison to the time needed for younger people.

The second equation of the SPM describes the hazard rate *μ*(*t*,**Y**(*t*)) (i.e., mortality/incidence rate) as a function of the stochastic covariates (see Eq. 3) [16, 21, 22]. The SPM represents the hazard as a quadtratic form: (**Y**(*t*)−**f**(*t*))^∗^ **Q**(*t*)(**Y**(*t*)−**f**(*t*)); hence it is also called the quadratic hazard model. Such a hazard form is a convenient and useful choice with acceptable statistical properties [17, 22] based on evidence that it is a quadratic function (J- or U-shape) of different covariates, see, e.g., [31–33, 35]. The hazard rate used in the model is also a function of time (age) and it also includes a baseline hazard *μ*_0_ which can be also time (age)-dependent (for example, Gompertz).

The parameter *Q*(*t*) controls how wide the U-shape (or a J-shape) is and can be formulated in terms of stress resistance [20, 40] or “vulnerability” [41]. As discussed in these works, robustness or vulnerability are characterized by the width of the U-shape, and, therefore, if the U-shape shrinks, the organism becomes more and more susceptible to deviations of biomarkers from their “normal” states. Such decreases of stress resistance can be indirectly captured from longitudinal data by the SPM.

SPM estimates physiological or biological *norms* of biomarker values which correspond to minimal hazard rates at some particular time (age) [42]: **f**(*t*). This is estimated explicitly since the quadratic term contains the difference between the biomarker value and some function denoting the *normal* (optimal) state: if a biomarker value **Y** equals the function **f** then the quadratic part is nullified. Any other values of **Y** not equal to **f** result in larger hazard rates. The difference **Y**−**f** also indicates that it was impossible for the organism to return to the *optimal state* and, therefore, the organism is deregulated.

### Application to blood glucose

Blood glucose (BG) has a tendency to increase with age and therefore to significantly differentiate from the normal level of BG determined among young adults. This can potentially contribute to increasing risks of death with age.

To study effects of BG on respective risks, researchers usually apply the Cox proportional hazards model. This gives one the estimates of coefficients *β* from which one can calculate the respective hazard ratios. Hazard ratios tell nothing about hidden and biologically interpretable components of aging processes, such as allostatic load, mean allostatic trajectory, stress resistance, adaptive capacity, and physiological norm. To see the effects of these components on mortality, which can not be captured by the Cox model, we performed analyses of repeated measurements of BG using SPM. This allows splitting the negative effects of external forces from the normal deterioration arising from the senescence process.

In this case study, we show that the level of BG which corresponds to the lowest mortality risk has a tendency to increase with age. The age-related changes in mortality-risk shape indicate the respective declines in stress-resistance which influence the level of BG. The case study results indicate that analyzing time-to-event data with SPM can substantially improve our knowledge of various factors and mechanisms which have an effect on aging-related changes in human organisms.

#### Data description

The Framingham Heart Study (FHS) Original Cohort was established in 1948 and has continued to the present [43]. In this study, we used the FHS data provided by the National Heart, Lung, and Blood Institute’s (NHLBI) Biologic Specimen and Data Repositories Information Coordinating Center (BioLINCC) resource (https://biolincc.nhlbi.nih.gov/home/). Version 2014a was used in the analyses. The dataset of *N* = 5,079 individuals (2,785 females, 2,294 males; almost all subjects are White/Caucasians). The minimum individual age is 28 years and the maximum is 104 years; the average age is 60.18 years. The average observational time was 12 years (6 exams) and the average time between consecutive observations was 2 years. Missing BG observations were removed from the analysis. A histogram of BG levels is given in Additional file 1: Figure S6.

#### Analysis methodology

We analyzed the data with R package stpm using a one-dimensional continuous-time model with time-dependent coefficients. Model parameters were in the form of linear functions and are presented below:

9 $$ \begin{aligned} \text{} & a(t) = a_{y} + b_{y} \cdot t \\ & f_{1}(t) = a_{f_{1}} + b_{f_{1}} \cdot t \\ & Q(t) = a_{q} + b_{q} \cdot t \\ & f(t) = a_{f} + b_{f} \cdot t \\ & b = const \\ & \mu(t) = \mu_{0} \cdot e^{\theta \cdot t} \end{aligned}  $$

Therefore, parameters *a*_*y*_, *b*_*y*_, $a_{f_1}$, $b_{f_1}$, *a*_*q*_, *b*_*q*_, *a*_*f*_, *b*_*f*_, *b*, *μ*_0_, *θ* were estimated.

#### Results

**Fig. 2:**
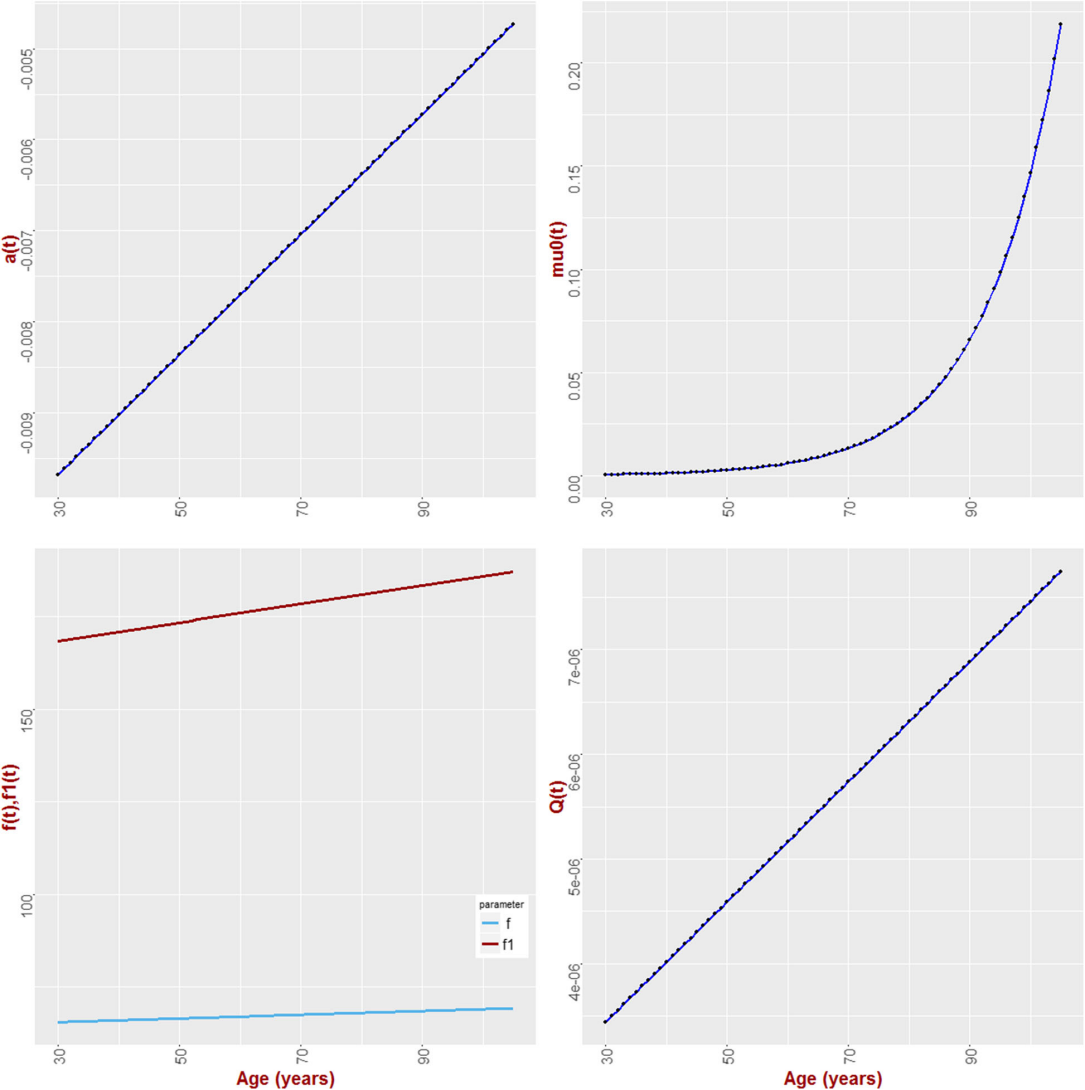
Model parameters *a*(*t*) (adaptive capacity of the organism), *f*_1_(*t*) (mean allostatic trajectory), *f*(*t*) (physiological norm - an optimal trajectory with minimum risk) *μ*_0_(*t*) (baseline hazard) and *Q*(*t*) (represents stress resistance)

The plots of *a*(*t*), *f*_1_(*t*), *f*(*t*), *Q*(*t*) and *μ*(*t*) are presented in Fig. 2. Numerical values of parameter estimates along with their statistical characteristics such as *s*.*d*. and confidence intervals are presented in Additional file 1: Table S3.

From Fig. 2 we can see that the value of BG changes with age as shown in [42]. The function **f**_1_ shows that the organism is not usually functioning in *normal* environment and therefore the trajectory of BG does not revert to the “norm” but rather to a different function. The increase with age in **Q**(*t*) indicates that the same deviation from the “norm” at older ages results in a larger increase in mortality risk. This means that the organism is more vulnerable to deviations of the level of BG from the normal value. We also see that the normal value is age-dependent. This indicates that the this optimal level of BG for younger individuals can actually increase the risk of death at older ages. Also, the age-dependence in **a**(*t*) shows that it takes more time for the trajectory of BG to go back to the allostatically prescribed value at older ages than it takes at younger ages. This means that the adaptive capacity of the organism (as related to adaptation to deviation of BG levels) declines with age.

As we show in this example, SPM [19] can estimate different aging-related components which eventually affect mortality though the longitudinal dynamics of physiological variables (such as BG). This provides a way to get additional insights into the processes of aging and serves as a background for further investigations using, for example, genetic analyses [26].

## Results

Tables 4, 5 and 6 show simulation results for one- and two-dimensional discrete-time SPM models with time-independent coefficients. Results for continuous-time SPM for both one- and two dimensions are provided in Additional file 1: Tables S1 and S2. All of the results show concordance with the parameter values used in simulation.

**Table 4:** Results of simulation studies for one-dimensional discrete-time model (5,000 individuals, 100 replications), estimated mean, standard deviation, lower and upper boundaries of empirical confidence interval (95th percentile) of estimated coefficients

Parameter	True	Est.mean	SD	LW	UP
a	-5.0000e-02	-5.0051e-02	1.1178e-03	-5.1884e-02	-4.8210e-02
f1	8.0000e+01	7.9966e+01	2.7216e-01	7.9619e+01	8.0390e+01
Q	1.0000e-06	1.0200e-06	8.4057e-08	8.8716e-07	1.1781e-06
f	8.0000e+01	7.9996e+01	9.4074e-02	7.9855e+01	8.0152e+01
b	5.0000e+00	4.9997e+00	1.0189e-02	4.9827e+00	5.0151e+00
mu0	1.0000e-05	1.0131e-05	1.5194e-06	8.3345e-06	1.2294e-05
theta	1.0000e-01	9.9750e-02	1.4026e-03	9.7000e-02	1.0200e-01

**Table 5:** Results of simulation studies for two-dimensional discrete-time model (5,000 individuals, 100 replications), estimated mean, standard deviation, lower and upper boundaries of empirical confidence interval (95th percentile) of estimated coefficients

Parameter	True	Est.mean	SD	LW	UP
a11	-5.0000e-02	-4.9908e-02	8.4712e-04	-5.0074e-02	-4.9742e-02
a12	1.0000e-03	9.3123e-04	4.2772e-04	8.4740e-04	1.0151e-03
a21	1.0000e-03	1.1607e-03	2.2296e-03	7.2369e-04	1.5977e-03
a22	-5.0000e-02	-5.0140e-02	9.9902e-04	-5.0336e-02	-4.9945e-02
f1 1	1.0000e+02	1.0071e+02	9.0962e+00	9.8931e+01	1.0250e+02
f1 2	2.0000e+02	1.9951e+02	4.4247e+00	1.9864e+02	2.0038e+02
Q11	1.0000e-06	1.0207e-06	1.2101e-07	9.9703e-07	1.0445e-06
Q12	1.0000e-07	1.0382e-07	3.7846e-08	9.6407e-08	1.1124e-07
Q21	1.0000e-07	1.0382e-07	3.7846e-08	9.6407e-08	1.1124e-07
Q22	1.0000e-06	1.0121e-06	7.8420e-08	9.9672e-07	1.0275e-06
f 1	1.0000e+02	1.0005e+02	3.6333e-01	9.9974e+01	1.0012e+02
f 2	2.0000e+02	2.0000e+02	1.8756e-01	1.9997e+02	2.0004e+02
b 1	2.0000e+00	2.0007e+00	3.7442e-03	2.0000e+00	2.0014e+00
b 2	5.0000e+00	4.9989e+00	8.4494e-03	4.9972e+00	5.0005e+00
mu0	1.0000e-04	1.0034e-04	8.5791e-06	9.8661e-05	1.0202e-04
theta	8.0000e-02	7.9900e-02	1.1237e-03	7.9680e-02	8.0120e-02

**Table 6:** Results of simulation studies for one-dimensional continuous-time model (5,000 individuals, 100 replications), with assuming time-dependent model coefficient *f*_1_=*f*_1*a*_+*f*_1*b*_ *t*, estimated mean, standard deviation, lower and upper boundaries of empirical confidence interval (95th percentile) of estimated coefficients

Parameter	True	Est.mean	SD	LW	UP
a	-5.0000e-02	-4.9620e-02	2.5252e-03	-5.3315e-02	-4.5373e-02
f1a	8.0000e+01	7.9899e+01	7.5520e-01	7.8839e+01	8.1205e+01
f1b	1.0000e-01	1.0196e-01	1.0402e-02	8.4886e-02	1.1978e-01
Q	1.0000e-05	1.0280e-05	5.1640e-06	1.3449e-06	1.8183e-05
f	8.0000e+01	7.7017e+01	2.4743e+01	3.0810e+01	1.1497e+02
b	2.5000e+00	2.4999e+00	2.1137e-02	2.4676e+00	2.5336e+00
mu0	1.0000e-01	9.6731e-02	4.6173e-03	8.4344e-02	1.0169e-01

We also provide histograms of estimated parameters for all model types. Figures S1-S5 from Additional file 1 show histograms of estimated parameter for one- and two-dimentional discrete-time models and the model with time-dependent coefficients.

## Discussion

The Stochastic Process Model allows researchers to utilize the full potential of longitudinal data by evaluating dynamic mechanisms of changing physiological variables with time (age), allowing the study of differences, for example, in genotype-specific hazards. Applying the Stochastic Process Model to analysis of longitudinal data can uncover influences of hidden components (adaptive capacity, allostatic load, resistance to stresses, physiological norm) of aging-related changes, which play important roles in aging-related processes but cannot be measured directly with common statistical methods. This provides researchers with a new way of analyzing longitudinal data.

The specific form of the hazard of risk function should be taken into account where conducting analyses of longitudinal data using SPM. In our approach, we assume that the hazard rate (incidence rate related to changing physiological variable with age) has a U- or J- shape, which is biologically justified by empirical observations. In reality, the true form of this function is not known and, since it is impossible to estimate the true form from the data, an incorrectly assumed hazard may introduce additional bias. Additional investigation is needed in order to evaluate the effects of different forms of hazard functions on results.

## Conclusion

We presented stpm - an R package that implements the Stochastic Process Model methodology. SPM can be used not only for stochastic modeling of probabilities of end-points but in many other applied areas, e.g., life science applications including biologically based modeling. In this work, the package was validated through simulation studies. The stpm R package can be extended by including: (i) SPM with several health states [29]; (ii) SPM with hidden heterogeneity [28]; (iii) SPM with competing risks [27]; and (iv) SPM for partially observed covariates [26].

## Availability and requirements

**Project name:** stpm **Project home page:** https://github.com/izhbannikov/spm/ **Operating systems:** Platform independent **Programming language:** R **Other requirements:** R 3.2.2 or higher + Rcpp, RcppArmadillo, mice, sas7bdat, stats, nloptr, survival, tools, knitr packages **Licence:** GPL licence

## Additional file

Additional file 1 **Supplementary materials.** **Table S1** Results of simulation studies for one-dimensional continuous-time model (5,000 subjects, 100 replications); Est.mean: estimated mean, SD: standard deviation, LW, UP: lower and upper boundaries of empirical confidence interval (95th percentile) of estimated coefficients. **Table S2** Results of simulation studies for two-dimensional continuous-time simulation (Var1 and Var2, 5,000 individuals, 100 replications); Est.mean: estimated mean; SD: standard deviation; LW, UP: lower and upper boundaries of empirical confidence interval (95th percentile) of estimated coefficients. **Figure S1** Histograms of estimated parameters of one-dimensional discrete-time model. Vertical red lines show the estimated means. Blue vertical lines indicate true parameters. **Figure S2** Histograms of estimated parameters of one-dimensional continuous-time model. Vertical red lines show the estimated means. Blue vertical lines indicate true parameters. **Figure S3** Histograms of estimated parameters of one-dimensional continuous-time model with time-dependent parameter f1 = f1a + f1bt; other parameters remained constant. Blue vertical lines indicate true parameters. Red vertical lines indicate estimated mean values of estimated parameters. **Figure S4** Histograms of estimated parameters for discrete-time two-dimensional model. Blue vertical lines indicate true parameters, red lines indicate estimated parameters. **Figure S5** Histograms of estimated parameters for continuous two-dimensional model. Blue vertical lines indicate true parameters, red lines indicate estimated parameters. **Table S3** Results of analysis Framingham Heart Study Data, Variable: blood glucose (BG); Est.mean: estimated mean; SD: standard deviation; LW, UP: lower and upper boundaries of empirical confidence interval (95th percentile) of estimated coefficients. There were 30 runs with different starting values of the model parameters. **Figure S6** Histograms of Blood Glucose (BG) level extracted from FHS original cohort. (760 KB DOCX)

## Abbreviations

BG: Blood glucose
BMI: Body mass index
DBP: Diastolic blood pressure
JM: Joint model
SPM: Stochastic process model
