# Supplementary material for: stpm: an R package for stochastic process model
Source: BMC Bioinformatics. 2017 Feb 23;18:125. doi: 10.1186/s12859-017-1538-7 (PMC5324240; doi:10.1186/s12859-017-1538-7)
Supplement: Supplementary file 1 — Supplementary materials. Table S1 Results of simulation studies for one-dimensional continuous-time model (5,000 subjects, 100 replications); Est.mean: estimated mean, SD: standard deviation, LW, UP: lower and upper boundaries of empirical confidence interval (95th percentile) of estimated coefficients. Table S2 Results of simulation studies for two-dimensional continuous-time simulation (Var1 and Var2, 5,000 individuals, 100 replications); Est.mean: estimated mean; SD: standard deviation; LW, UP: lower and upper boundaries of empirical confidence interval (95th percentile) of estimated coefficients. Figure S1 Histograms of estimated parameters of one-dimensional discrete-time model. Vertical red lines show the estimated means. Blue vertical lines indicate true parameters. Figure S2 Histograms of estimated parameters of one-dimensional continuous-time model. Vertical red lines show the estimated means. Blue vertical lines indicate true parameters. Figure S3 Histograms of estimated parameters of one-dimensional continuous-time model with time-dependent parameter f1 = f1a + f1bt; other parameters remained constant. Blue vertical lines indicate true parameters. Red vertical lines indicate estimated mean values of estimated parameters. Figure S4 Histograms of estimated parameters for discrete-time two-dimensional model. Blue vertical lines indicate true parameters, red lines indicate estimated parameters. Figure S5 Histograms of estimated parameters for continuous two-dimensional model. Blue vertical lines indicate true parameters, red lines indicate estimated parameters. Table S3 Results of analysis Framingham Heart Study Data, Variable: blood glucose (BG); Est.mean: estimated mean; SD: standard deviation; LW, UP: lower and upper boundaries of empirical confidence interval (95th percentile) of estimated coefficients. There were 30 runs with different starting values of the model parameters. Figure S6 Histograms of Blood Glucose (BG) level extracted from FHS original co [file 12859_2017_1538_MOESM1_ESM.docx]

Supplementary materials

# A: Simulation studies

## One – dimensional simulation

We simulated 100 follow-up databases with discrete intervals between the observations (1 year), with N = 5,000 subjects in each database, with one physiological variable (covariate). Trajectory projections were performed according to the methodologies described in Akushevich et al. (2005).

Separately, we simulated another set of 100 follow-up databases for continuous-time model (arbitrary intervals between consecutive observations) and model with time-dependent parameters; see Yashin et al. (2007b). For the test of the continuous-time model all parameters remained constant. For the test of the model with time-dependent coefficients we set parameter *f*_1_ to be time-dependent (*f*_1_ = *f*_1_*_a_* + *f*_1_*_b_t*); other parameters remained constant.

**Table S1** Results of simulation studies for one-dimensional continuous-time model (5,000 subjects, 100 replications); Est.mean: estimated mean, SD: standard deviation; LW, UP: lower and upper boundaries of empirical confidence interval (95^th^ percentile) of estimated coefficients.

|  | **True** | **Est.mean** | **SD** | **LW** | **UP** |
| --- | --- | --- | --- | --- | --- |
| a | -1.0000e-01 | -1.0009e-01 | 7.4994e-04 | -1.0139e-01 | -9.8898e-02 |
| f1 | 8.0000e+01 | 7.9999e+01 | 2.1359e-02 | 7.9967e+01 | 8.0033e+01 |
| Q | 1.0000e-06 | 9.7888e-07 | 1.5711e-07 | 6.5343e-07 | 1.1926e-06 |
| f | 8.0000e+01 | 8.0146e+01 | 1.2895e+00 | 7.7979e+01 | 8.2356e+01 |
| b | 1.0000e+00 | 9.9999e-01 | 1.5602e-03 | 9.9698e-01 | 1.0022e+00 |
| mu0 | 1.0000e-05 | 9.3150e-06 | 1.4928e-06 | 6.6664e-06 | 1.1785e-05 |
| theta | 8.0000e-02 | 8.0678e-02 | 1.8045e-03 | 7.8061e-02 | 8.3965e-02 |

## Two-dimensional simulation

We also simulated 100 datasets (with discrete (1 year) and arbitrary intervals between observations) of 5,000 individuals, using two physiological variables (Var1 and Var2).

**Table S2** Results of simulation studies for two-dimensional continuous-time simulation (Var1 and Var2, 5,000 individuals, 100 replications); Est.mean: estimated mean; SD: standard deviation; LW, UP: lower and upper boundaries of empirical confidence interval (95^th^ percentile) of estimated coefficients.

|  | **True** | **Est.mean** | **SD** | **LW** | **UP** |
| --- | --- | --- | --- | --- | --- |
| a11 | -1.0000e-01 | -9.8497e-02 | 4.6600e-03 | -1.0783e-01 | -9.2229e-02 |
| a12 | 1.0000e-03 | 1.0197e-03 | 7.4298e-05 | 9.1836e-04 | 1.1483e-03 |
| a21 | 1.0000e-03 | 1.0528e-03 | 7.4681e-05 | 9.4230e-04 | 1.1712e-03 |
| a22 | -1.0000e-01 | -9.6912e-02 | 4.6476e-03 | -1.0663e-01 | -9.0792e-02 |
| f1_1 | 1.0000e+02 | 1.0111e+02 | 3.2798e+00 | 9.5817e+01 | 1.0699e+02 |
| f1_2 | 2.0000e+02 | 1.9970e+02 | 2.4438e+00 | 1.9545e+02 | 2.0354e+02 |
| Q11 | 1.0000e-06 | 1.0480e-06 | 6.5178e-08 | 9.5919e-07 | 1.1578e-06 |
| Q12 | 1.0000e-07 | 1.0471e-07 | 6.5960e-09 | 9.5024e-08 | 1.1513e-07 |
| Q21 | 1.0000e-07 | 1.0324e-07 | 5.7843e-09 | 9.3734e-08 | 1.1342e-07 |
| Q22 | 1.0000e-06 | 1.0301e-06 | 6.5376e-08 | 9.3171e-07 | 1.1297e-06 |
| f_1 | 1.0000e+02 | 1.0080e+02 | 3.3344e+00 | 9.5825e+01 | 1.0611e+02 |
| f_2 | 2.0000e+02 | 2.0050e+02 | 3.2181e+00 | 1.9629e+02 | 2.0609e+02 |
| b_1 | 1.0000e+00 | 9.8283e-01 | 8.3901e-02 | 8.5759e-01 | 1.1404e+00 |
| b_2 | 2.0000e+00 | 2.0056e+00 | 1.1352e-01 | 1.8293e+00 | 2.1937e+00 |
| mu0 | 1.0000e-04 | 1.0232e-04 | 5.8479e-06 | 9.2445e-05 | 1.1069e-04 |
| theta | 8.0000e-02 | 8.0609e-02 | 1.5870e-03 | 7.7941e-02 | 8.2573e-02 |

## Histograms of estimated parameters

Presented below are the histograms of estimated parameters for discrete- and continuous-time models for one variable, from simulated data. The histograms show that in general the distributions of the parameter estimates are close to normal distributions.


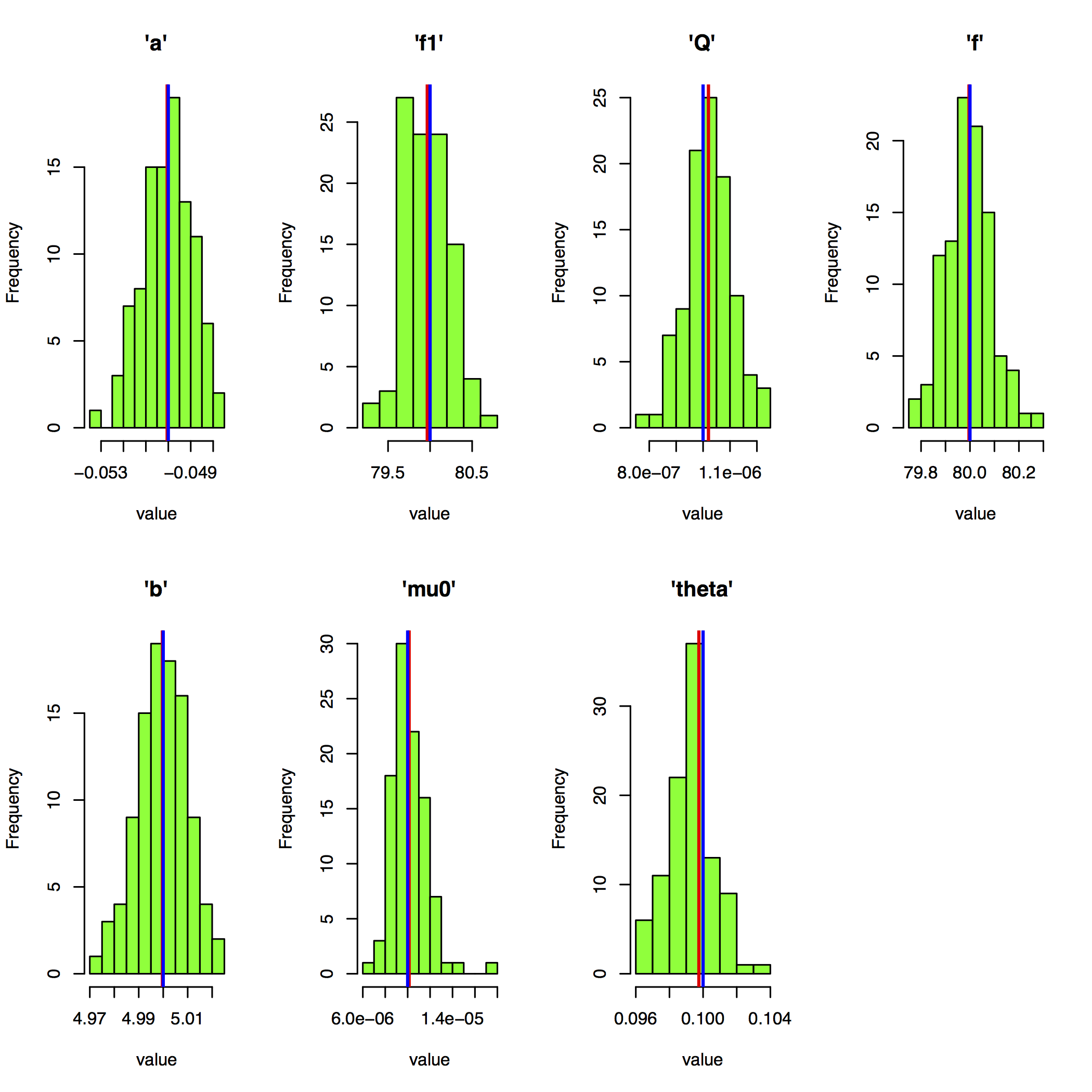


**Figure S1** Histograms of estimated parameters of one-dimensional discrete-time model. Vertical red lines show the estimated means. Blue vertical lines indicate true parameters.


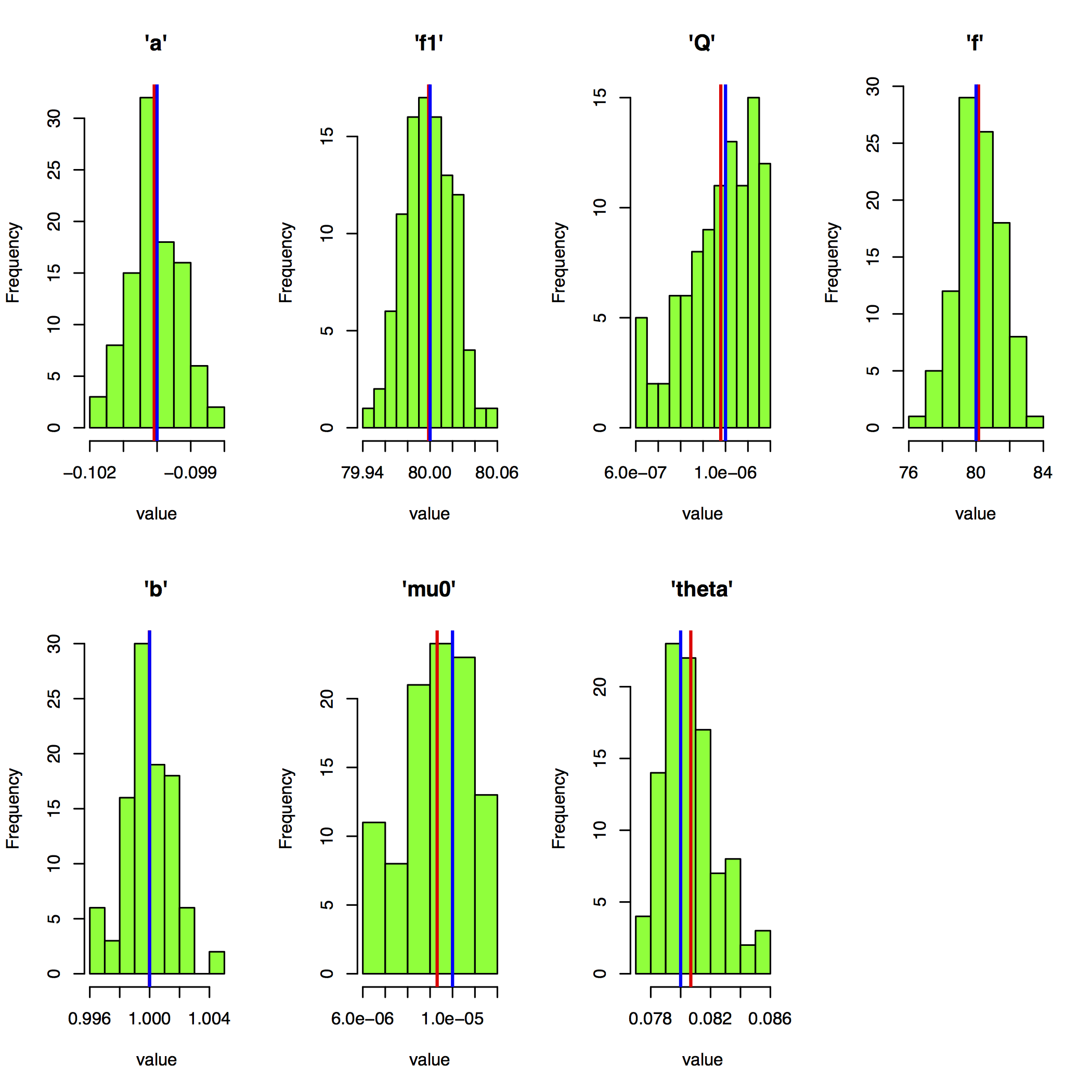


**Figure S2** Histograms of estimated parameters of one-dimensional continuous-time model. Vertical red lines show the estimated means. Blue vertical lines indicate true parameters.


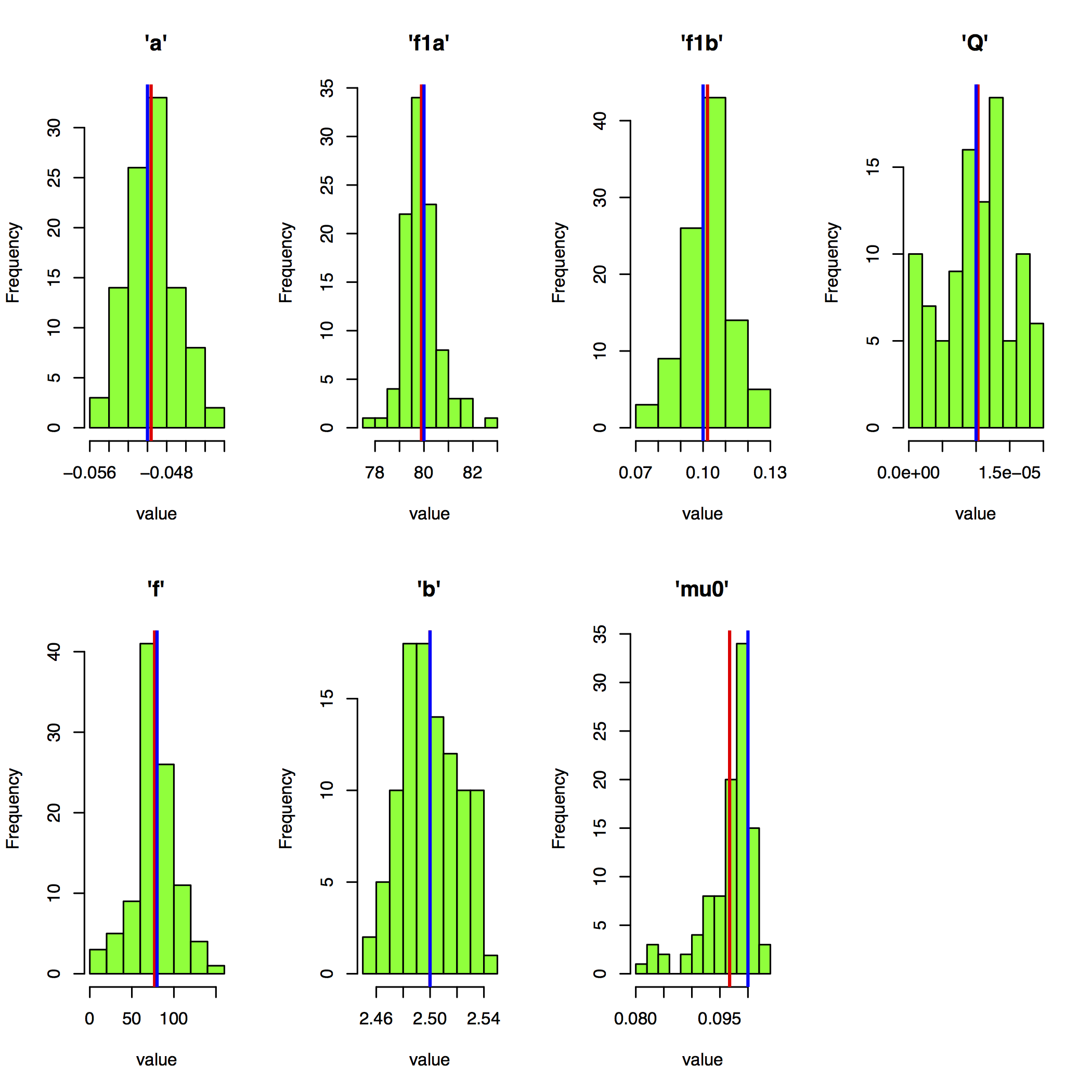


**Figure S3** Histograms of estimated parameters of one-dimensional continuous-time model with time-dependent parameter *f*_1_ *= f*_1_*_a_ + f*_1_*_b_t*; other parameters remained constant. Blue vertical lines indicate true parameters. Red vertical lines indicate estimated mean values of estimated parameters.


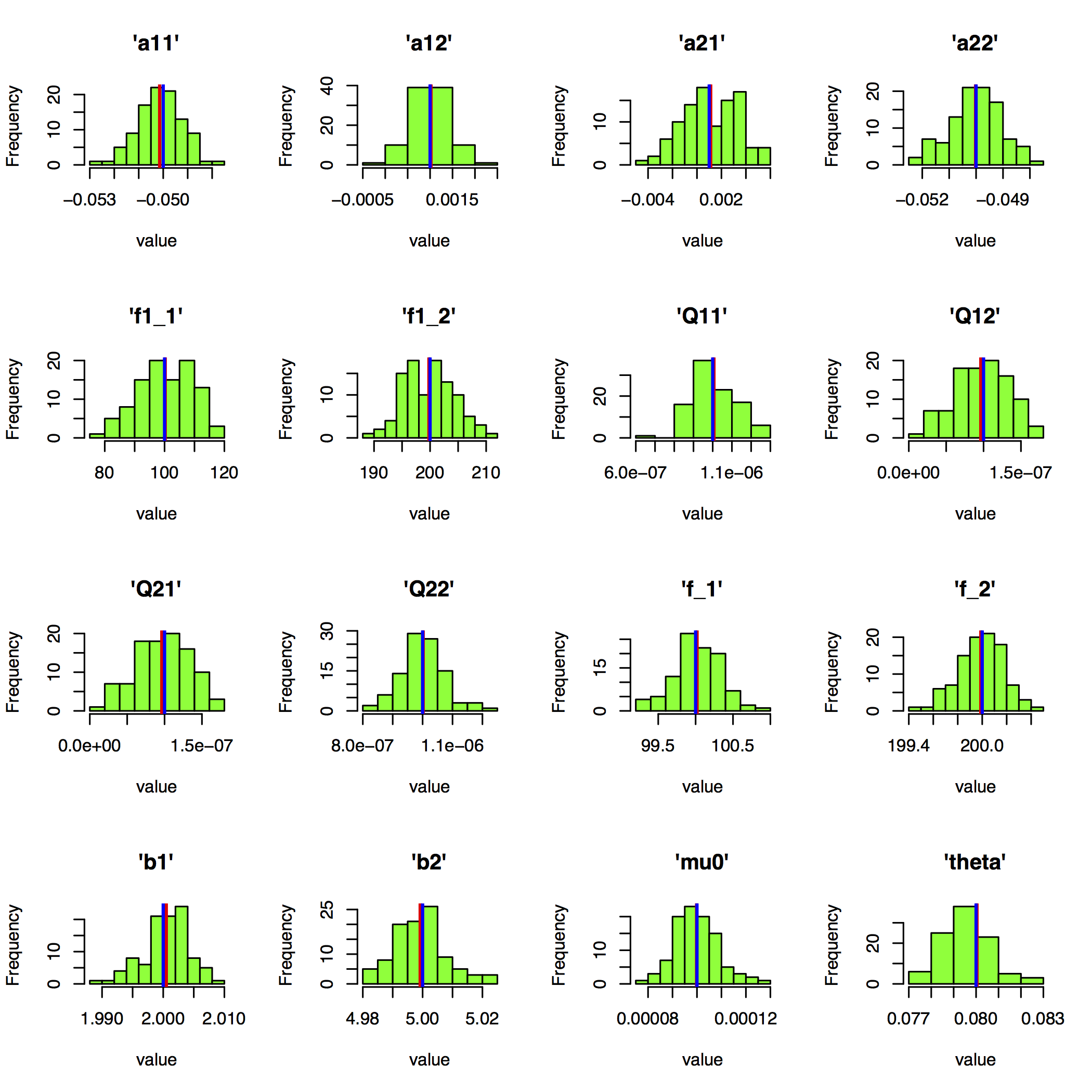
 **Figure S4** Histograms of estimated parameters for discrete-time two-dimensional model. Blue vertical lines indicate true parameters; red lines indicate estimated parameters.


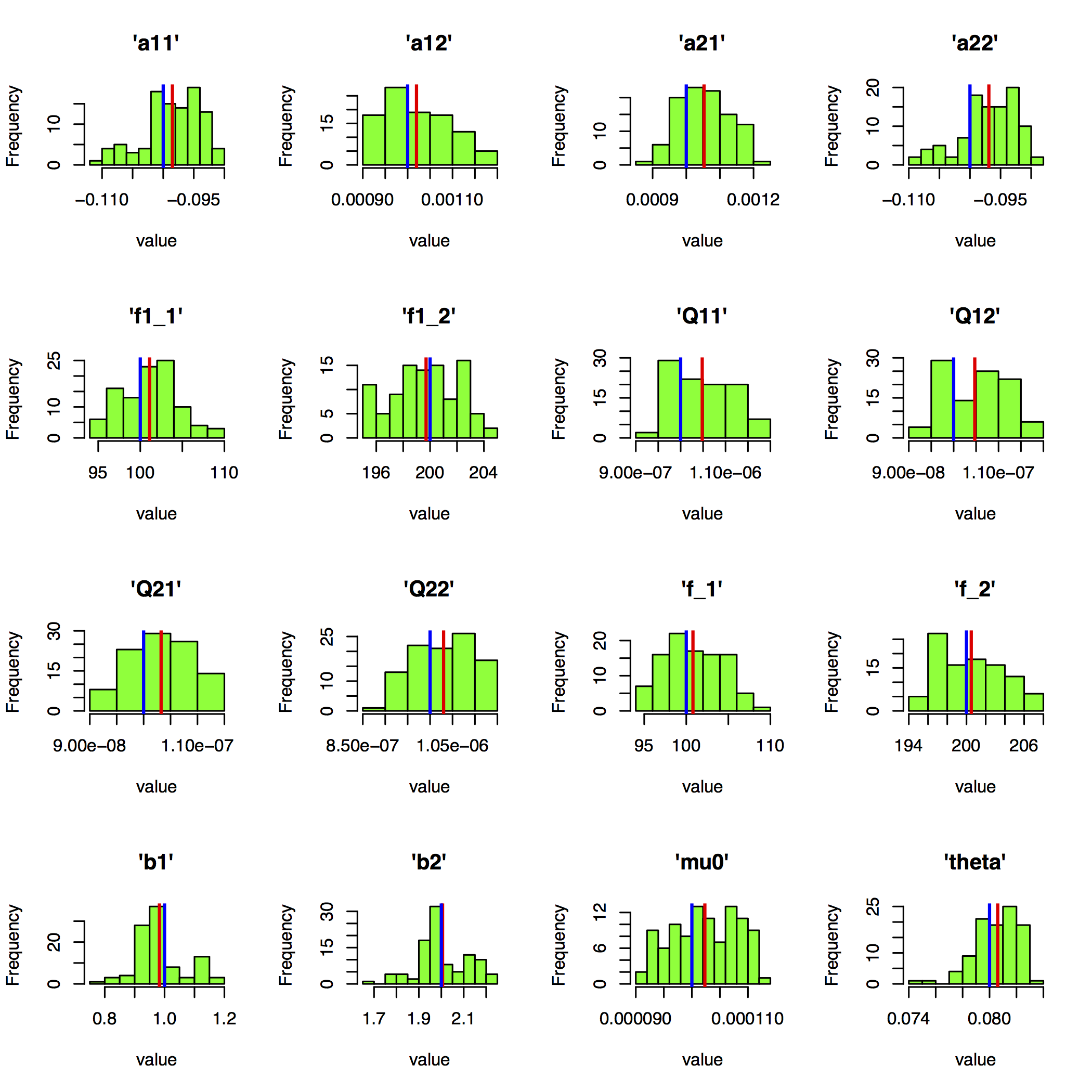


**Figure S5** Histograms of estimated parameters for continuous two-dimensional model. Blue vertical lines indicate true parameters; red lines indicate estimated parameters.

# B: Numerical methods used in implementation of the algorithms

## Discrete-time model (Akushevich et al, 2005)

To access the parameters we used a generalized linear model with family *Binomial* and link *Log* (for calculating *mu0*, ***b***, ***Q*** and *theta* in Gompertz function) and a linear auto-regression (for calculating ***u***, ***R*** and *Sigma*).

## Continuous-time model (Yashin et al, 2007a)

For calculation of *m(t)* and *γ(t)* from corresponding differential equations we used the standard Runge-Kutta method (“RK4”).

For calculation of the survival function, we used Simpson’s method with variable number of steps, from 2 to 2*(t2-t1) if (t2-t1) > 2.

# C: Case study: application to Framingham Heart Study (original cohort) data, blood glucose (BG)

**Table S3** Results of analysis of Framingham Heart Study Data, Variable: blood glucose (BG); Est.mean: estimated mean.

| **Parameter** | **Est.mean** |
| --- | --- |
| **ay** | -1.1654e-02 |
| **by** | 6.5936e-05 |
| **af1** | 1.6097e+02 |
| **bf1** | 2.5000e-01 |
| **aq** | 1.7185e-06 |
| **bq** | 5.7411e-08 |
| **af** | 6.4061e+01 |
| **bf** | 5.0000e-02 |
| **b** | 9.1714e+00 |
| **mu0** | 4.9208e-05 |
| **theta** | 7.9998e-02 |


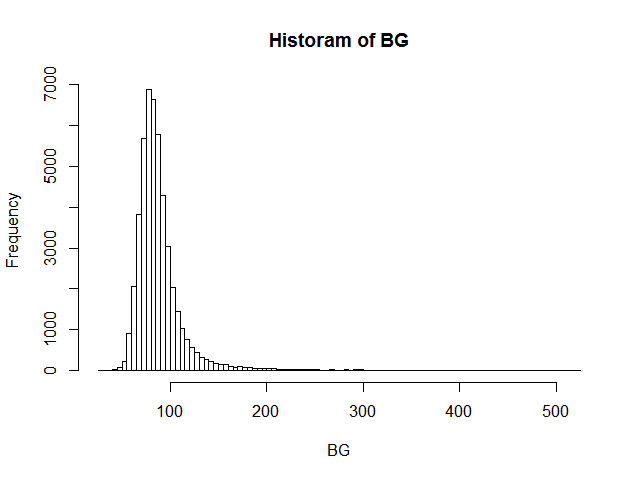


**Figure S6** Histograms of Blood Glucose (BG) level extracted from FHS original cohort.

## References

Akushevich I., Kulminski A. and Manton K.: Life tables with covariates: Dynamic model for Nonlinear Analysis of Longitudinal Data. 2005. Mathematical Population Studies, 12(2), pp.: 51-80.

Yashin, A.I. et al (2007a). Stochastic model for analysis of longitudinal data on aging and mortality. Mathematical Biosciences, 208(2), 538 – 551.

Yashin, A. et al (2007b). Health decline, aging and mortality: how are they related? Biogerontology, 8(3), 291–302.
